# Immune‐Mediated Necrotizing Myopathy Diagnosis and Flare in an Adolescent After COVID‐19 Vaccine

**DOI:** 10.1002/ccr3.71287

**Published:** 2025-10-20

**Authors:** Patricia Hoffman, Ezra Chefitz, Cynthia Salvant, Daniel B. Horton

**Affiliations:** ^1^ Department of Pediatrics Rutgers Robert Wood Johnson Medical School New Brunswick New Jersey USA; ^2^ Hospital for Special Surgery Pediatric Rheumatology Fellowship New York New York USA; ^3^ University of Pittsburgh Medical Center, Pediatric Gastroenterology Fellowship Pittsburgh Pennsylvania USA; ^4^ Albany Medical Center Albany New York USA

**Keywords:** COVID‐19, inflammatory myopathy, pediatrics, rheumatology, vaccine

## Abstract

We discuss a teenager who developed a rare pediatric autoimmune muscle disease after completing the primary COVID‐19 vaccine series, which flared shortly after COVID‐19 booster vaccination. This case suggests that a rare chronic pediatric muscle disease may represent a possible adverse event related to COVID‐19 vaccination.

## Graphical abstract

Muscle biopsy is histopathologically consistent with a diagnosis of immune‐mediated necrotizing myopathy.

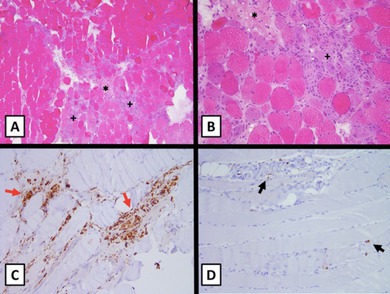

**Abbreviations**
anti‐HMGCR: anti‐3‐hydroxy‐3‐methylglutarly coenzyme A reductase
anti‐SRP: anti‐signal recognition particle
anti‐SSA: anti‐Sjogren's Syndrome type A antigen
CK: creatine phosphokinase
COVID‐19: coronavirus disease 2019
EBV: epstein–barr virus
IMNM: immune‐mediated necrotizing myopathy
JDM: juvenile dermatomyositis
JIIMs: juvenile idiopathic inflammatory myopathies
MRI: magnetic resonance imaging
RF: rheumatoid factor

## 1 Introduction

Juvenile idiopathic inflammatory myopathies (JIIMs) are a group of heterogeneous autoimmune disorders affecting skeletal muscle, resulting in acute or subacute onset of symmetric progressive proximal muscle weakness and elevated muscle enzymes [1]. JIIMs are rare, with an incidence between 1.6 and 4 cases per million children per year and a prevalence of 2.5 cases per 100,000 children [2]. The most common type of JIIM is juvenile dermatomyositis (JDM) (80% of JIIMs); other JIIMs include amyopathic dermatomyositis, inclusion body myositis, polymyositis, and immune‐mediated necrotizing myopathy (IMNM) [1]. The etiology of JDM is unclear, but it may result from an autoimmune reaction triggered by environmental factors in genetically susceptible individuals [1].

## 2 Case History and Examination

A 15‐year‐old female presented to the pediatric emergency department in October 2021 with 2 months of worsening muscle weakness, calf pain, and limited range of arm motion. Other recent symptoms included right hip stiffness, color changes in the fingertips with cold exposure, increasing fatigue, and decreased appetite, along with an unintentional 10‐pound weight loss over the past month. Previously active in multiple sports, she was no longer able to participate in any sport and required assistance with activities of daily living. She denied fevers, chills, rashes, difficulty swallowing, or bowel or bladder incontinence. She had received mRNA coronavirus disease 2019 (COVID‐19) vaccine (Pfizer/BioNTech) earlier that year, with the second vaccination (June 2021) occurring approximately 7 weeks before the reported onset of symptoms. She had no known recent exposures to COVID‐19 or mononucleosis.

On initial physical exam, she had normal vital signs and reduced strength in proximal muscles (3/5 at shoulders and hips, 4/5 at elbows and knees, and 5/5 at wrists and ankles). She walked slowly and was unable to rise from a chair without using her arms. The rest of the physical exam, including skin exam, was unremarkable. Initial bloodwork revealed marked elevations in creatine phosphokinase (CK) (28,138), aspartate aminotransferase (928), alanine transaminase (619), lactate dehydrogenase (3152), and urinary myoglobin (> 8750). Inflammatory markers were elevated (C‐reactive protein 4.68, erythrocyte sedimentation rate 39), and blood cell counts were normal.

## 3 Differential Diagnosis

The patient's presentation was concerning for viral myositis, rhabdomyolysis, or JIIM. A magnetic resonance imaging (MRI) of the right lower extremity revealed extensive proximal intermuscular and sub‐fascial edema, which was consistent with the expected findings seen in JIIM. Additional testing revealed positive antinuclear antibodies (1:1280 titer, cytoplasmic pattern), positive anti‐Sjogren's Syndrome antigen A (SSA/Ro), and positive rheumatoid factor (RF). Additional history‐taking revealed a recent increase in dental caries and dry mouth, without dry or burning eyes or a history of cheek swelling, concerning for Sjogren's disease. Epstein Bar Virus (EBV) VCA IgG was positive, but EBV IgM and cytomegalovirus PCR were negative. Aldolase was markedly elevated (> 360). She did not have any unexplained fevers, hair loss, oral or nasal ulcers, or joint swelling or stiffness. She did not have recent medication use to suggest drug‐induced myopathy. Muscular dystrophies were considered unlikely based on her age and sex.

## 4 Outcome and Follow‐Up

On hospital day 8, the patient experienced sudden‐onset chest pain and hemoptysis. Computerized tomography angiogram of the chest revealed a pulmonary embolus. Electrocardiogram, echocardiogram, and high‐resolution chest computed tomography were all normal. Pulmonary function testing showed a mixed obstructive and restrictive pattern with normal diffusion capacity for carbon monoxide. MRI/magnetic resonance venogram of the abdomen showed compression of the left common iliac vein, consistent with a diagnosis of May‐Thurner Syndrome. Enoxaparin was initiated.

**FIGURE 1:**
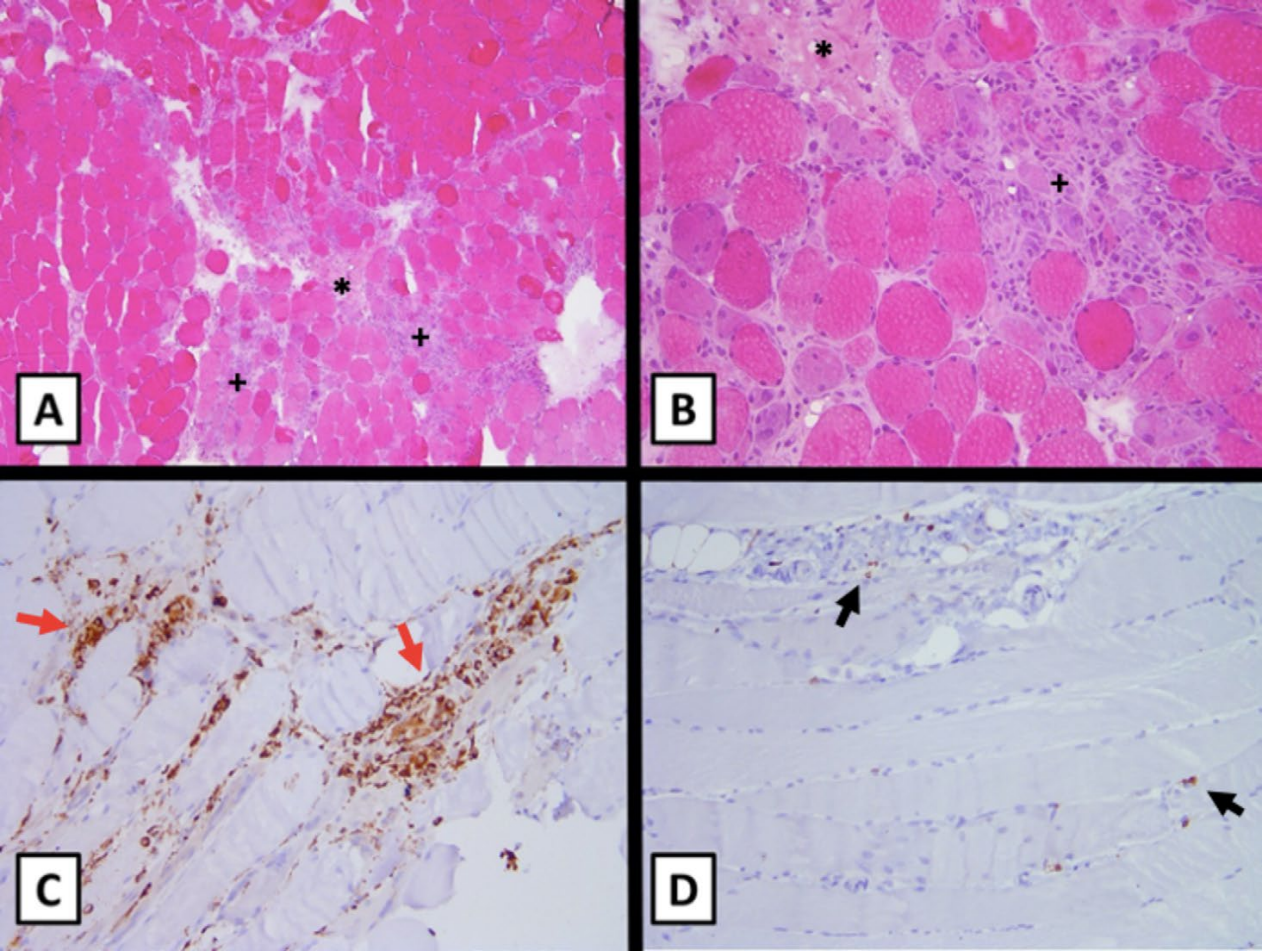
Muscle biopsy. Hemotoxylin and eosin stained cryosections magnified 100× (A) and 200× (B) show several necrotic (asterisk) and regenerating muscle fibers (cross). Paraffin section‐immunohistochemistry illustrates numerous CD68(+) macrophages (C, thin/red arrows) and rare perivascular CD3(+) T‐cells (D, thick/black arrows) within the necrotic fibers.

**FIGURE 2:**
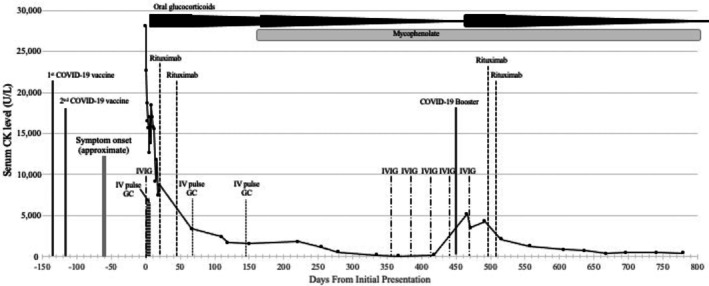
Clinical timeline. CK levels (solid line, units/l) and key events through the first 2 years of illness. Events include COVID‐19 vaccination (double line), approximate symptom onset (gray line), IVIG (long dash‐dotted line), IV pulse glucocorticoids (GC) (dotted line), rituximab (dashed line), oral glucocorticoids (black bar), and mycophenolate (gray bar).

The patient's muscle biopsy of the right thigh revealed necrotic fibers with limited inflammatory cells, suggestive of IMNM (Figure 1). Additional testing showed high‐titer anti‐signal recognition particle (anti‐SRP) antibody (1:61,440) and negative anti‐3‐hydroxy‐3‐methylglutaryl coenzyme A reductase (anti‐HMGCR), consistent with a diagnosis of IMNM (Figure 2). She was treated with intravenous immunoglobulin (IVIG) and pulse solumedrol, followed by rituximab and prolonged oral prednisone taper (Figure 2). She was transferred to an inpatient rehabilitation center 22 days after hospitalization with a rolling walker.

Two months after hospital discharge, she developed chest pain and cough with blood‐tinged mucus and tested positive for COVID‐19 for the first time. Repeat CK was increased, but she did not experience worsening muscle weakness and continued with the scheduled prednisone taper. Nonetheless, continued CK elevations prompted initiation of mycophenolate mofetil (later switched to mycophenolic acid due to gastrointestinal intolerance) and an increase in prednisone dose, followed by monthly IVIG infusions. On this regimen, her muscle strength improved, she regained the ability to walk, the CK normalized, and prednisone was again tapered. However, within 1 week after receiving her COVID‐19 booster, she experienced worsening fatigue and rapidly worsening proximal muscle weakness, with renewed difficulty lifting her legs and arms, rising from a seated position, and swallowing. There were no symptoms or signs of recurrent COVID‐19. Repeat CK in the emergency room was 6311 (up from 167 pre‐booster), and she received another pulse of methylprednisolone. She did not require hospitalization at that time. Oral prednisone was increased to 60 mg daily, and she started another course of rituximab soon thereafter. Subsequent lung imaging revealed subpleural reticulation, compatible with interstitial lung disease.

## 5 Discussion

JIIM occurs during childhood and presents with weakness, chronic inflammation of skeletal muscles, and, for JDM and amyopathic dermatomyositis, skin changes [3]. Autoantibodies are important for identifying phenotypes, guiding monitoring and treatment, and offering prognosis [3]. Myositis‐specific autoantibodies are present in about 60% of children with JIIIM and are associated with disease manifestations [2]. Muscle biopsy can be useful in confirming or refuting the JIIM diagnosis when there is an atypical presentation, and MRIs aid in diagnosis and surveillance [1].

While IMNM is the second most common form of IIM in adults, it is rare among JIIMs [1, 4]. The damage from muscle necrosis in IMNM leads to the release of more muscle enzymes than in other types of IIM, reflected by generally higher CK levels [4]. Anti‐SRP and anti‐HMGCR are both associated with IMNM and detected rarely in JIIM (1.6%–2% and 1%, respectively) [1]. SRP is a ubiquitous ribonuclear protein that regulates protein translocation across the endoplasmic reticulum [5]. Compared to those with anti‐HMGCR, those with anti‐SRP tend to have extramuscular manifestations, including dysphagia, Raynaud phenomenon, and interstitial lung disease, all of which affected our patient [6]. Muscle biopsy in IMNM is characterized by necrotic myofibers with few, if any, MHC class 1 inflammatory cells [7].

Viral infections, including EBV, human immunodeficiency virus, and COVID‐19, are well‐described causes of acute myositis [8]. While our patient had evidence of prior EBV infection, she had no associated clinical features suggesting that EBV or SARS‐CoV‐2 triggered her myositis, and her condition was chronic. While autoimmune myositis in adults, including IMNM, is often associated with malignancies, cancer‐associated myositis is rare in children, and routine malignancy screening is not recommended after JIIM diagnosis [1].

A link has been postulated between COVID‐19 vaccines and the development of autoimmune myositis in adults. Several cases of anti‐SRP positive IMNM have been reported in adults (ages 26–71) who received the primary COVID‐19 vaccination, with weakness beginning 7–26 days after the second dose [9, 10, 11, 12, 13] and, in one case, shortly after the first dose [14]. In a review of 49 published cases of myositis associated with COVID‐19 vaccination, mean age was 56 ± 17 years (no pediatric cases), 59% were in females, 70% followed mRNA vaccines (often after the second dose), and only 3 were associated with anti‐SRP antibodies [15]. One UK study reported a temporal relationship of new autoimmune myositis diagnoses following either RNA or DNA COVID‐19 vaccines, more commonly following the second vaccination [16]. In a systematic review of 74 published studies of adverse reactions or events related to mRNA COVID‐19 vaccination, systemic adverse events more commonly occurred following the second vaccination and within 12 weeks (or 84 days) [17], which is consistent with our patient's presentation. These studies, like the case presented here, lend credence to the possibility that autoimmune myositis may, in some susceptible individuals, represent a vaccine‐related adverse event.

Still, little is known about COVID‐19 vaccination and autoimmune myositis in children. One 16‐year‐old girl newly diagnosed with JDM was reported to develop symptoms within hours after the first mRNA COVID‐19 vaccination, a rapid time course suggesting the unmasking of subclinical disease rather than the triggering of a new illness [18]. In south central Europe, new pediatric autoimmune diagnoses were more common after COVID‐19 than after COVID‐19 vaccination [19]. One of these reported cases was acute (not chronic) myositis, occurring in a 16‐year‐old girl months following COVID‐19 and within hours after a second mRNA COVID‐19 vaccine [20]. In contrast, our patient's symptoms began many weeks after vaccination, arguing against (though not definitively disproving) the hypothesis that her disease was unmasked by the vaccine.

Our patient had an uncommon phenotype (anti‐SRP‐positive IMNM) of a rare pediatric family of diseases (JIIM), with symptomatic onset within 2 months following the second COVID‐19 vaccination of the primary series and a flare within 1 week after the first vaccine booster. Neither her diagnosis nor flare followed SARS‐CoV‐2 infection, and an intercurrent episode of COVID‐19 was not accompanied by clinically significant worsening of her disease. The patient's initial diagnosis of IMNM was not originally attributed to the earlier vaccination. While causality cannot be confirmed in this case, the timing of her subsequent post‐booster flare (within 1 week after booster vaccination), along with other literature linking COVID‐19 vaccines with chronic autoimmune myositis in adults, suggests the possibility of a causal connection in our patient.

Our patient did not have clinical features suggesting an undifferentiated connective tissue disease. However, she did present with several manifestations of Sjogren's disease, including dry mouth, recent caries, and positive testing for anti‐SSA and RF, although the time of onset of these symptoms relative to COVID‐19 vaccination was unclear. Anti‐SRP IMNM has been reported in adults with Sjogren's disease [21]. Additionally, one case of IMNM following COVID‐19 vaccination has been reported in an adult previously diagnosed with Sjogren's disease [22]. Sjogren's disease and IMNM are both rare in pediatric populations and have never been reported to co‐occur in children, suggesting that our patient may have had a genetic predisposition to autoimmune disease independent of the role of COVID‐19 vaccination.

Based on COVID‐19 vaccination recommendations from the American College of Rheumatology, patients receiving immunosuppressants for rheumatic diseases are recommended to receive COVID‐19 vaccine boosters [19]. Multiple studies have investigated COVID‐19 vaccine safety in patients with autoimmune myositis. In one study, patients with IMNM had a lower risk of reported adverse events after COVID‐19 vaccines compared to other autoimmune conditions, particularly after the Pfizer/BioNTech vaccine [23]. In another study of children with pre‐existing JIIMs, most did not experience flares after COVID‐19 vaccination or COVID‐19 [24].

Given its rarity, there is no standardized treatment approach for IMNM in children, which is frequently managed with recommended treatments for adults with IMNM, including glucocorticoids and steroid‐sparing therapies, including IVIG and rituximab [25, 26].

## 6 Conclusion

We report an adolescent girl who developed progressive symptoms of a rare chronic muscle disease, immune‐mediated necrotizing myopathy, within 2 months following completion of the primary COVID‐19 mRNA vaccination series and who flared within 1 week following booster vaccination. This patient also had clinical features of Sjogren's disease, another rare pediatric condition that has not previously been reported to co‐occur with IMNM in children. This unique case raises the hypothesis that the COVID‐19 vaccine may, in rare cases, trigger autoimmune muscle disease in susceptible children. More research is needed to better understand the potential mechanistic link between COVID‐19 vaccination and autoimmune myositis as a possible vaccine‐related adverse event.

## Author Contributions

**Patricia Hoffman:** conceptualization, data curation, writing – original draft, writing – review and editing. **Ezra Chefitz:** conceptualization, data curation, writing – review and editing. **Cynthia Salvant:** conceptualization, supervision, writing – review and editing. **Daniel B. Horton:** supervision, writing – review and editing.

## Consent

The authors confirm the patient's and the patient's parent's written consent have been signed and collected.

## Conflicts of Interest

The authors declare no conflicts of interest.

## Data Availability

Data sharing not applicable to this article as no datasets were generated or analyzed during the current study.
